# The Philadelphia Polyclinic

**Published:** 1896-09

**Authors:** 


### Editorial, Etc.

### THE PHILADELPHIA POLYCLINIC.

From time to time this Journal has printed selections from the *Philadelphia Polyclinic* which is edited by a committee of the faculty. The "Case of removal of the Major Portion of the Lower Jaw for Sarcoma" in the July number of this Journal was reported by the Professor of Surgery in the Philadelphia Polyclinic and college for graduates in medicine. This institution was organized in December, 1882, and opened for clinical work and teaching early in 1883. It has steadily extended its facilities and opportunities for practical work, until now it has a corps of fifty professors, lecturers and adjunct professors and thirty-one instructors. It makes a feature of offering direct personal instruction and opportunities for actual clinical work.

The Polyclinic opens to all graduates in medicine its facilities for acquiring a practical acquaintance with the clinical aspects of disease and the diagnostic procedure and methods of treatment in general medicine and surgery that were formerly attained only by those who obtained positions as resident physicians in the larger hospitals. The members of its limited classes personally examine cases of disease and employ the instruments of precision in diagnosis, participating in the actual work of the clinic and the laboratory.

A Roentgen apparatus is daily used for diagnosis and study; valuable researches in the department of Neuro-Pathology have been published; the Infirmary service represents rare and obscure diseases sent by physicians from a distance; the number of major operations performed at the Polyclinic Hospital during the past year was over 500, including abdominal sections.

As a cordial invitation has been extended to inspect the institution, the readers of the Journal who visit Philadelphia may find it profitable to study the model arrangements.

